# Emotional intelligence as a mediator in the relationship between academic performance and burnout in high school students

**DOI:** 10.1371/journal.pone.0253552

**Published:** 2021-06-24

**Authors:** María del Mar Molero Jurado, María del Carmen Pérez-Fuentes, África Martos Martínez, Ana Belén Barragán Martín, María del Mar Simón Márquez, José Jesús Gázquez Linares

**Affiliations:** 1 Department of Psychology, Faculty of Psychology, University of Almería, Almería, Spain; 2 Department of Psychology, Faculty of Psychology, Universidad Politécnica y Artística del Paraguay, Asunción, Paraguay; 3 Department of Psychology, Universidad Autónoma de Chile, Providencia, Chile; Universtiyt of Oviedo (Spain), SPAIN

## Abstract

Low performance of high school students and repeating a year are major problems in the education system. Low performance in the classroom generates negative emotions in young people and has been related to development of school burnout. The objective of this study was to analyze the repercussions of academic performance on burnout in high school students, and establish the role of emotional intelligence in this relationship. The sample was made up of 1287 high school students aged 14 to 18, who filled out questionnaires for evaluation of these variables. The results showed that youths who had failed a subject or had repeated a year showed more exhaustion and cynicism than their classmates with better performance and higher academic efficacy. A relationship was also found between school burnout and emotional intelligence in these adolescents, positive for self-efficacy and negative for cynicism and exhaustion. The model results showed that low academic performance affected burnout level, and that stress management and mood in emotional intelligence acted as a mediator in this relationship. In conclusion, development of emotional intelligence programs in the educational context is proposed as a measure for preventing burnout in the face of adverse high school academic events, such as failing or repeating a year.

## Introduction

### Academic performance and burnout

Academic performance is one of the major problems in the Spanish education system [1]. As shown by data from the Program for International Student Assessment (PISA Report), Spain is below the mean performance of the rest of the countries in the Organization for Economic Cooperation and Development (OECD). And although the number of repeaters has gone down in recent years, the mean is still much higher than the rest of the OECD [2].

These data cause strong concern due to the high risk that students with low performance will leave school [2, 3]. Furthermore, low academic risk in high school has been related to other risks, such as school absenteeism [3, 4], low academic motivation [4, 5] and educational aspirations, and poorer levels of self-efficacy [6, 7], lower subjective wellbeing [8], drinking alcohol and taking drugs [3, 8, 9] or being victimized by cyberbullying [10]. Its influence on participation and commitment of adolescents in the school has been demonstrated [4, 11, 12]. And in the long term, academic problems related to lower academic performance and repeating a year have been associated with less likelihood of a higher education and precarious employment [13].

Along with performance, the appearance of student burnout has been studied [7]. Academic exhaustion, or burnout syndrome, appears as a result of high levels of school stress and is characterized by the student feeling continually overwhelmed, attributing little value to school and feeling detached, not enjoying school activities [14]. Burnout develops when students are subjected to stress for long periods and pressured to reach goals at school, affecting their aspirations and beliefs about their own performance [7]. This can be divided into three dimensions: exhaustion, meaning emotional fatigue when faced with continual demands; cynicism (that is, indifferent, distant attitude to school); and efficacy (expectations about the ability to reach academic goals successfully) [15]. This syndrome, along with school participation, have been found to be the two factors involved in school wellbeing [16, 17]. Furthermore, it means students have a more negative perspective of the future [18], and is a risk factor for developing behaviors such as smoking [19], altered sleep quality and symptoms of depression, anxiety and stress linked with negative affect [20].

The relationship between performance and school burnout was studied by Palos et al. [17], who evaluated classroom performance by means of grades, and found that it was an antecedent for developing burnout, but not the opposite. Along this line, Engels, Pakarinen, Lekkanen & Verschueren [21], in a longitudinal study, demonstrated that higher levels of exhaustion did not lead to lower performance in young people, although it did find a negative relationship between the two variables. Nevertheless, there are few studies analyzing how student achievement can affect school burnout, as performance is usually employed as a measure of results and not as an antecedent variable [21]. This study was intended to add to evidence on the relationship between performance and burnout, expecting to find that there are differences in burnout factors depending on academic performance of adolescents.

### Emotional intelligence (EI) in the school: Influence on school burnout

Adolescence is characterized by experiencing very intense emotions [22], so emotional intelligence has been confirmed as a very influential construct in educational environments [23]. This variable has been described as the capability of individuals to recognize and discriminate their own and others’ emotions, using this information to guide thinking and behavior [24]. Findings on emotional intelligence show that emotions are functional when the information they provide is discerned, interpreted precisely and managed effectively. Thus, when emotional intelligence is high, the emotional response to an event is accompanied by cognitive and behavioral changes that make the individual adaptive [23, 25]. Emotional intelligence of students, that is, how they feel faced with academic events, such as repeating a year or failing an exam, and how they use and respond to their feelings influences the school setting, supporting wellbeing and development or the complete opposite [26]. And along this line, use of the new technologies has a fundamental role in developing new digital tools for integrating emotional content in the learning process, promoting socioemotional competencies, wellbeing and adaptation in adolescence [27].

In a recent study on feelings that high school students experience about the school, it was found that three out of four emotions were negative, regardless of the sociodemographic characteristic of the students. The most frequent were tiredness, stress and boredom [28]. In turn, unpleasant emotions in the school context have been associated with negative consequences, such as school burnout [29]. In other cases, an association has been found between social anxiety and low academic performance, with the possibility that the first could affect the second by limiting concentration in the classroom [30].

Emotional intelligence makes it possible to be aware of, manage and cognitively handle negative emotions, minimizing the anxiety, confusion and stress that inevitably arise from life events and in adolescence, thereby, avoiding the appearance of cynicism or depersonalization, key symptoms of burnout [31, 32]. Thus, emotional intelligence training programs have shown to be effective in reducing burnout and emotional symptoms, and at the same time increase life satisfaction and self-esteem of the participants [33]. Similar results have been found in improving wellbeing and reducing burnout and school stress in young students [34–37]. Therefore, the second hypothesis of this study was that there is a negative correlation between school burnout and the components of emotional intelligence, such as stress management and mood.

Academic emotions, on the other hand, are related to school processes, and are different in young people with high or low academic performance. High performance has been related to more positive student emotions, such as satisfaction, calm, relief, enjoyment, hope and pride, while low performance is associated with prevalence of feelings such as anger, anxiety, shame, hopelessness, boredom, depression, distress or exhaustion [37]. In addition, students with low academic performance seem to regulate their emotions worse than those with high performance, impeding school stress management [38]. This may be partly because young people with high levels of emotional intelligence are better able to cope with situations at school, feel self-realization and satisfaction with tasks performed and effort made [39].

They also enjoy and feel more connected to the school, since they have better relations with classmates and teachers than those who have low emotional intelligence [40], because they regulate anxiety during interaction better [41], which could counteract negative emotional states that arise at school. On this basis, this study expected to find factors such as the stress management or mood components of emotional intelligence to act as mediators in the relationship between academic performance and burnout, facilitating a more positive emotional perspective and management of negative feelings and the distress that appears when classroom performance is low, and thereby reducing the probability of students developing burnout symptoms.

### This study

The school is one of the central elements in young people’s lives during adolescence, and determines part of their wellbeing [17]. According to Fiorilli et al. [4], inquiry into the factors involved in school adjustment can contribute to improving the wellbeing of students during both their school years and adulthood, more so because burnout in students has negative implications for their quality of life as adults. Therefore, the objective of this study was to analyze the differences in school burnout by academic performance and examine the involvement of variables related to EI in the relationship between academic performance and burnout, in a sample of high school students. Our research hypotheses were:

H1: There are significant differences in the factors of burnout according to student academic performance
H2: There is a negative correlation between school burnout and EI components such as stress management and mood.
H3: Stress management and mood are the components of EI that mediate in the relationship between academic performance and burnout.

## Method

### Participants

First, cluster sampling was used to select the schools. Eleven high schools in the province of Almeria participated, all of them in urban areas. Participants were selected by simple random sampling.

A total of 1287 students at public high schools () in the province of Almería (Spain), aged 14 to 18 with a mean age of 15.11 (*SD* = 0.91) participated in this study. The sex distribution was 47.1% (*n* = 606) boys and 52.9% (*n* = 681) girls, with mean ages of 15.12 (*SD* = 0.94) and 15.10 (*SD* = 0.88), respectively.

### Instruments

An ad hoc questionnaire was prepared. Three questions to collect student sociodemographic data (age, sex, grade) and two questions on their academic performance were asked to find out whether they had ever failed a subject (“*Have you ever failed a subject*?”) and whether they had ever repeated a year, (“*Have you ever repeated a year*?”). In both cases, the answer choice was dichotomous (yes/no).

The Maslach Burnout Inventory-Survey (MBI-SS) [14] validation for Spanish students [42] was used for evaluating school burnout. This instrument contains 12 items rated on a seven-point Likert scale (where 1 is “never” and 6 is “every day”). The items are grouped in three subscales: emotional exhaustion, which refers to the feeling of being physically, mentally and emotionally tired of school (e.g.: “I feel emotionally exhausted by my schoolwork.”), cynicism, which refers to self-criticism and loss of interest in schoolwork (e.g.: “I am less interested in studying since I started high school”) and academic self-efficacy, understood as feelings of academic competence (e.g., I can solve the problems that come up at school effectively”). The reliability indices for each of the subscales were *ω* = 0.83 for exhaustion, *ω* = 0.82 in cynicism and *ω* = 0.79 in self-efficacy.

The *Brief Emotional Intelligence Inventory* (EQ-i-M20) [43] was used for evaluation of emotional intelligence. This tool consists of 20 items which enable five factors to be evaluated: intrapersonal (e.g., “I can describe my feelings easily”), interpersonal, (e.g., “I understand well how other people feel”), stress management (e.g., “It is hard for me to control my anger”), adaptability (e.g., “I can solve problems different ways”) and general mood (e.g., “I am happy with the kind of person I am”). These factors refer to the capability for understanding one’s own feelings and those of others, the ability for self-control in distressing situations, flexibility in problem solving and feeling happy and optimistic, respectively. The answer choices are on a Likert-type scale from 1 “never happens to me”) to 4 (“always happens to me”). The reliability of the instrument for this study was *ω* = 0.81 for the Intrapersonal factor, *ω* = 0.62 for Interpersonal, *ω* = 0.77 in Stress management, *ω* = 0.71 for Adaptability and *ω* = 0.87 in Mood.

### Procedure

Before collecting the information, the school principals were contacted to inform them of the objectives of the study, and guarantee confidential data processing. Two members of the research team went to the schools to administer the questionnaires. First, they gave the students the appropriate instructions and guaranteed the anonymity of their answers. The students filled out the tests individually, in an estimated mean time of 25–30 minutes. In all cases, in compliance with ethical research standards, all participants accepted voluntary participation and had the written consent of the parents / guardians at the center for their participation. The study was approved by the University of Almería Bioethics Committee (Ref: UALBIO2018/015).

### Data analysis

First, to find out whether there were any between-group differences in the dimensions of school burnout by performance (had not failed a subject vs had failed a subject, and had not repeated a year vs repeated a year), a Student’s *t-*test for independent samples was carried out. In this case, the Bayesian alternative was computed to be able to compare the evidence of the alternative hypothesis (H_1_: there are between-group differences) with the null hypothesis (H_0_: there are no between-group differences). JASP version 0.11.1 [44] statistical software was used for estimation of the Bayesian *t*-test. The Cauchy prior width was set at the software default (r = 0.70), which means there is a 70% probability that the real effect size is between -0.5 and 0.5.

To find out the distribution and identify the relationship between emotional intelligence and school burnout, Pearson’s bivariate correlation analysis was applied. The emotional intelligence components that correlated to the dimensions of school burnout were extracted from the results in the correlation matrix.

Then the mediation analysis was computed, where academic performance data (fail a subject/repeat a year) were the predictors, the emotional intelligence components were mediators, and the school burnout dimensions were the response variables. JASP mediation analysis based on lavaan software [45] was used for computing the models. Models were estimated by applying bootstrapping and the confidence intervals were calculated using the bias-corrected percentile method, following Biesanz, Falk & Savalei [46].

To examine the reliability of the instruments used for data collection, McDonald’s Omega [47] coefficient was estimated, following the proposal and guidelines by Ventura-León & Caycho [48].

## Results

### Burnout and academic performance (fail a subject)

Table 1 shows the significant differences between those who had failed a subject and those who had not. Specifically, those who had failed had significantly higher scores in exhaustion (*t*_(1284)_ = -3.20, *p* < 0.01, d = -0.21) and cynicism (*t*_(1284)_ = -6.03, *p* < 0.001, *d* = -0.41), while those who had never failed a subject had a higher mean score in self-efficacy (*t*_(1284)_ = 3.67, *p* < 0.001, *d* = 0.74).

**Table 1:** Descriptive statistics and Bayesian independent samples *t*-test (failed subject).

						95% CI (MD)		BF₁₀	error %
	Group	N	Mean	SD	MD	Lower	Upper		
EXH	no	274	13.51	6.37	-1.33	-2.157	-0.520	11.749	1.791e -6
	yes	1008	14.85	6.05					
CYN	no	274	8.23	6.37	-2.63	-3.489	-1.777	3.647e +6	1.692e -11
	yes	1008	10.86	6.41					
EFF	no	274	17.37	4.38	3.67	3.013	4.340	1.281e +23	1.072e -27
	yes	1008	13.69	5.11					

*Note*. EXH = Exhaustion, CYN = Cynicism, EFF = Self-efficacy. SD = Standard deviation, MD = Mean difference, BF = Bayes Factor.

For exhaustion, the BF_10_ = 11.749, which shows that the data observed were 11.74 times more likely under H_1_ than H_0_. Cynicism had a BF_10_ showing that observed data were 3.647 x 10^6^ times more likely under H_1_ than H_0_. For self-efficacy, the BF_10_ showed that observed data were 1.281 x 10^23^ times more likely under H_1_ than under H_0_.

**Fig 1:**
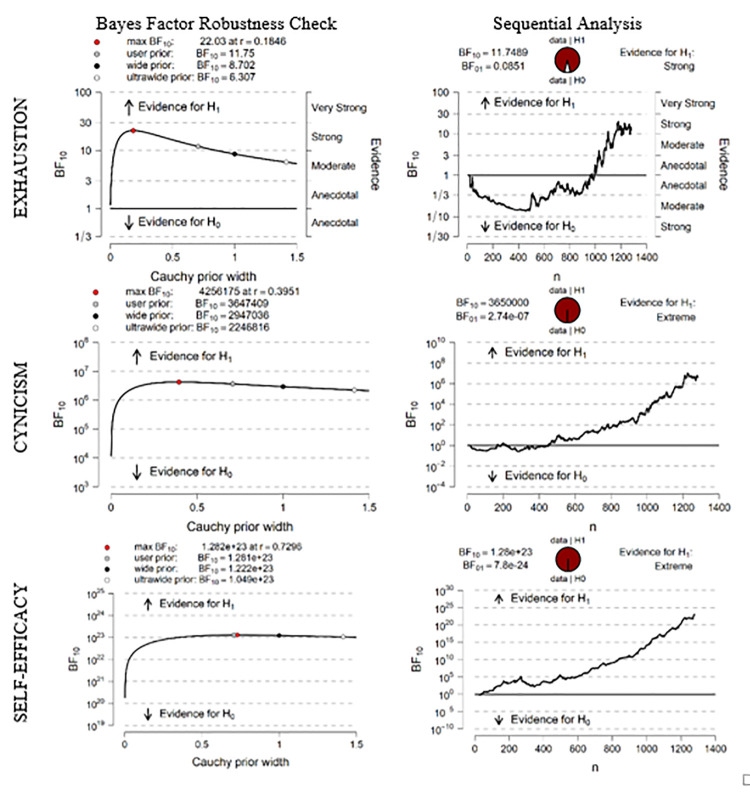
Inferential plots (fail subject).

In Fig 1, the first column shows the results of the robustness check and the second, sequential analyses combined with the Bayes Factor robustness check.

### Burnout and academic performance (repeat year)

As shown in Table 2, repeaters had the highest scores in cynicism (*t*_(1284)_ = -3.53, *p* < 0.001, *d* = -0.23). However, those who had never repeated a year had a higher mean score in self-efficacy (*t*_(1284)_ = 4.52, *p* < 0.001, *d* = 0.29). No statistically significant between-group differences were observed in exhaustion (*t*_(1284)_ = -0.49, *p* = 0.624).

**Table 2:** Descriptive statistics and Bayesian independent samples *t*-test (repeat year).

						95% CI (MD)		BF₁₀	error %
	Group	N	Mean	SD	MD	Lower	Upper		
EXH	no	976	14.52	6.16	-0.19	-0.982	0.589	0.082	0.006
	yes	310	14.71	6.06					
CYN	no	976	9.94	6.52	-1.49	-2.320	-0.665	33.585	1.076e -5
	yes	310	11.44	6.29					
EFF	no	976	14.85	5.05	1.51	0.860	2.177	1611.919	1.887e -7
	yes	310	13.33	5.43					

*Note*. EXH = Exhaustion, CYN = Cynicism, EFF = Self-efficacy. SD = Standard deviation, MD = Mean difference, BF = Bayes Factor.

For exhaustion, the Bayesian approach was more easily interpreted in terms of BF_01_. In this case, the data observed were 12.17 times more likely under H_0_ than under H_1_. For cynicism, the BF_10_ showed that data were 33.58 times more likely under H_1_ than under H_0_. Finally, for self-efficacy, the BF_10_ showed that the data observed were 1612 times more likely under H_1_ than H_0_.

**Fig 2:**
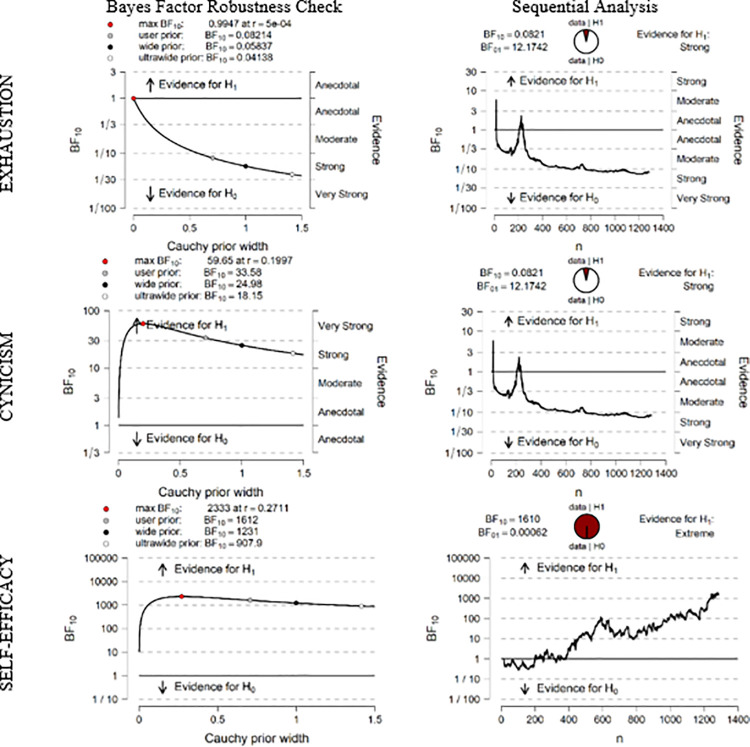
Inferential plots (repeat year).

In Fig 2, the first column shows the results of the robustness check and the second column the sequential analyses combined with the Bayes Factor robustness check.

### School burnout and emotional intelligence

As observed in the correlation matrix (Table 3), exhaustion was negatively related to the intrapersonal components, stress management and mood. The correlation was positive with the interpersonal factor, although intensity was weak. Cynicism correlated negatively with the intrapersonal factors, stress management, adaptability and mood. For self-efficacy, the correlations were positive with all the emotional intelligence components.

**Table 3:** School burnout and emotional intelligence. Bivariate correlations.

		Intrapersonal	Interpersonal	Stress management	Adaptability	Mood
Exhaustion	Pearson’s r	-0.090**	0.065*	-0.242***	0.007	-0.195***
	Upper 95% CI	-0.036	0.119	-0.190	0.062	-0.142
	Lower 95% CI	-0.144	0.010	-0.293	-0.048	-0.247
Cynicism	Pearson’s r	-0.075**	0.009	-0.238***	-0.066*	-0.177***
	Upper 95% CI	-0.020	0.063	-0.186	-0.011	-0.124
	Lower 95% CI	-0.129	-0.046	-0.289	-0.120	-0.230
Self-efficacy	Pearson’s r	0.161***	0.190***	0.121***	0.323***	0.330***
	Upper 95% CI	0.214	0.242	0.175	0.371	0.378
	Lower 95% CI	0.107	0.137	0.067	0.273	0.280

*Note*. * p < 0.05

** p < 0.01

*** p < 0.001.

### Analysis of the mediating effect of emotional intelligence in the relationship between academic performance and school burnout

Table 4 shows the direct effects of having failed a subject on the three school burnout components. The analysis of indirect effects revealed the existence of a mediating effect of stress management, which intervened in the relationship between failing and school burnout (exhaustion, cynicism and self-efficacy). While adaptability was found to be a mediator with self-efficacy as the response variable. The total effects of the model were significant for all of the dimensions of school burnout.

**Table 4:** Direct, total and indirect effects (fail subject).

Direct effects					95% Confidence Interval	
	Estimate	Std. Error	z-value	p	Lower	Upper
Fail → EXH	0.152	0.066	2.303	0.021	0.027	0.283
Fail → CYN	0.320	0.066	4.862	< 0.001	0.197	0.447
Fail → EFF	-0.591	0.061	-9.691	< 0.001	-0.697	-0.477
Indirect effects					95% Confidence Interval	
	Estimate	Std. Error	*z*-value	*p*	Lower	Upper
Fail → INTRA → EXH	-0.001	0.004	-0.245	0.807	-0.015	0.007
Fail → INTER → EXH	-0.008	0.006	-1.306	0.192	-0.028	6.642e -4
Fail → STR-M → EXH	0.068	0.017	4.081	<0 .001	0.042	0.107
Fail → ADAPT → EXH	-0.015	0.010	-1.440	0.150	-0.039	0.005
Fail → MOOD → CYN	0.022	0.012	1.835	0.067	9.748e -4	0.048
Fail → INTRA → CYN	-6.683e -4	0.003	-0.243	0.808	-0.012	0.004
Fail → INTER → CYN	-0.005	0.004	-1.095	0.273	-0.024	6.336e -4
Fail → STR-M → CYN	0.067	0.017	4.064	< 0.001	0.041	0.103
Fail → ADAPT → CYN	0.006	0.010	0.619	0.536	-0.016	0.029
Fail → MOOD → CYN	0.017	0.010	1.767	0.077	0.002	0.040
Fail → INTRA → EFF	3.372e -4	0.001	0.234	0.815	-0.003	0.009
Fail → INTER → EFF	-0.007	0.005	-1.272	0.203	-0.025	5.875e -4
Fail → STR-M → EFF	-0.023	0.009	-2.422	0.015	-0.049	-0.006
Fail → ADAPT → EFF	-0.058	0.015	-3.791	<0 .001	-0.092	-0.031
Fail → MOOD → EFF	-0.030	0.016	-1.890	0.059	-0.063	-7.526e -4
Total effects					95% Confidence Interval	
	Estimate	Std. Error	z-value	p	Lower	Upper
Fail → EXH	0.218	0.068	3.210	0.001	0.083	0.351
Fail → CYN	0.406	0.067	6.039	< 0.001	0.267	0.522
Fail → EFF	-0.709	0.065	-10.874	< 0.001	-0.818	-0.592

*Note*. EXH = Exhaustion, CYN = Cynicism, EFF = Self-efficacy, INTRA = Intrapersonal, INTER = Interpersonal, STR-M = Stress management, ADAPT = Adaptability, MOOD = Mood. [Delta method standard errors, bias-corrected percentile bootstrap confidence intervals].

Table 5 shows the results of the mediation analysis taking having repeated a year as the antecedent variable. In this case, direct effects of repeating the year were found on cynicism and self-efficacy. The analysis of indirect effects revealed that stress management is the emotional intelligence factor which could be mediating in the relationship between repeating a year and school burnout, and this path is significant for the three components (exhaustion, cynicism and self-efficacy). The total effects of the model were significant for cynicism and self-efficacy.

**Table 5:** Direct, total and indirect effects (repeat year).

Direct effects					95% Confidence Interval	
	Estimate	Std. Error	z-value	p	Lower	Upper
Repeat → EXH	-0.026	0.062	-0.420	0.674	-0.144	0.097
Repeat → CYN	0.168	0.063	2.682	0.007	0.051	0.289
Repeat → EFF	-0.244	0.059	-4.115	<0 .001	-0.374	-0.117
Indirect effects					95% Confidence Interval	
	Estimate	Std. Error	z-value	p	Lower	Upper
Repeat → INTRA → EXH	0.004	0.004	0.933	0.351	-0.002	0.018
Repeat → INTER → EXH	-0.007	0.006	-1.231	0.218	-0.025	0.002
Repeat → STR-M → EXH	0.060	0.016	3.740	<0 .001	0.031	0.104
Repeat → ADAPT → EXH	-0.002	0.003	-0.727	0.467	-0.016	0.001
Repeat → MOOD → CYN	0.003	0.011	0.311	0.756	-0.018	0.024
Repeat → INTRA → CYN	0.002	0.003	0.750	0.453	-0.001	0.016
Repeat → INTER → CYN	-0.004	0.004	-1.061	0.289	-0.019	8.197e -4
Repeat → STR-M → CYN	0.058	0.016	3.708	< 0.001	0.031	0.101
Repeat → ADAPT → CYN	0.002	0.003	0.761	0.447	-0.002	0.016
Repeat → MOOD → CYN	0.003	0.009	0.311	0.756	-0.014	0.020
Repeat → INTRA → EFF	-3.928e -4	0.002	-0.205	0.838	-0.009	0.003
Repeat → INTER → EFF	-0.006	0.005	-1.181	0.238	-0.020	0.002
Repeat → STR-M → EFF	-0.024	0.009	-2.680	0.007	-0.049	-0.009
Repeat → ADAPT → EFF	-0.013	0.014	-0.919	0.358	-0.046	0.012
Repeat → MOOD → EFF	-0.005	0.015	-0.311	0.756	-0.034	0.025
Total effects					95% Confidence Interval	
	Estimate	Std. Error	z-value	p	Lower	Upper
Repeat → EXH	0.032	0.065	0.491	0.623	-0.098	0.149
Repeat → CYN	0.230	0.065	3.540	<0 .001	0.104	0.347
Repeat → EFF	-0.293	0.065	-4.527	<0 .001	-0.425	-0.160

*Note*. EXH = Exhaustion, CYN = Cynicism, EFF = Self-efficacy, INTRA = Intrapersonal, INTER = Interpersonal, STR-M = Stress management, ADAPT = Adaptability, MOOD = Mood. [Delta method standard errors, bias-corrected percentile bootstrap confidence intervals].

## Discussion

Low performance of high school students and repeating a year are the problems most affecting education in Spain [2]. Beyond the implications for young people’s own professional development [4, 13], low academic performance has been found to be a variable related to the appearance of other risks [3, 5, 8, 9, 10] impeding adolescent adjustment, such as school burnout [7]. As school adjustment is an important variable in adolescence as well as in the long-term, in adulthood [4], the objective of this study was to inquire into some of the factors involved, and particularly, to analyze the repercussion of low academic performance on burnout symptoms in high school students and the role of emotional intelligence in this relationship.

Previous studies have shown the existence of associations between student performance and school burnout symptoms [7, 21], the first established as an antecedent of classroom burnout [17]. On this basis, the first hypothesis was proposed postulating the existence of significant differences in burnout factors by academic performance. Our findings confirmed that young people who have failed a subject are more exhausted and cynical, while those who had not failed showed more self-efficacy. And similarly, students who had repeated a year showed more cynicism and less self-efficacy than their classmates who were not repeaters. Adolescents who repeated a year were more cynical about the value of education and felt less able to undertake schoolwork successfully, like those who had failed a course and also felt emotionally overwhelmed.

Positive relationships were also found between the self-efficacy factor of burnout and all the emotional intelligence factors. Significant negative relationships of cynicism and exhaustion were found with intrapersonal emotional intelligence, stress management and mood, where the strongest relationship was with the last two factors. These results confirm the second hypothesis of our study and show how recognition of one’s own feelings, their proper management, especially when overwhelming, and favorable mood, are associated with lower incidence of school burnout symptoms linked to continual stress and emotional exhaustion [31, 33, 35].

Finally, a model was found that confirmed the mediator effect of the stress management and mood factors in the relationship between performance and school burnout, supporting the third hypothesis proposed. These results sustain that low academic performance, defined as failing a subject and repeating a year, affect the development of student burnout symptoms through stress management and mood. Thus, when adolescents show low levels in these emotional intelligence factors, situations that generate distress, stress and negative feelings, such as failing or repeating a year, can lead to the appearance of this syndrome. In this line, other authors have suggested that as most of the feelings about school that appear during adolescence are negative [28], especially in young people with low performance [37]. These emotions, if not recognized, controlled and efficiently managed, can derive in maladaptive responses to stress and distress, such as school burnout [26].

In view of all of the above, working on emotional competences in the school is confirmed as an effective measure for protecting from development of exhaustion and lowering its levels in secondary education [27, 34–37], protecting young people from this syndrome and its consequences, especially among those with performance levels below expected. According to Szczygiel and Mikolajczak [49], the protective role of emotional intelligence in developing burnout has scarcely been analyzed. However, findings in other groups point in the same direction as the results of our study, showing the buffer role of trait emotional intelligence in the relationship between stressful, adverse situations and burnout, promoting better management [49].

This study had some limitations. First, the construction of a wider theoretical argument to back the results was hindered by the low number of studies analyzing performance as an antecedent variable of school burnout. In future, it would be of interest to continue research in this direction, linking psychological symptoms and syndromes, such as burnout with mood caused by adverse situations in the school. Furthermore, factors such as the number of subjects failed or academic expectations that could be affecting stress and distress experienced by the participants were not considered. As school burnout is a syndrome that develops from maintaining high levels of stress, future research should include these and other variables that could be affecting young people’s mood toward their academic situation.

## Conclusions

Adolescents spend a large part of their time at school, making it one of the major elements in their lives, and therefore, marking to a great extent their present and future wellbeing. During a stage where the individual is easily influenced by a multitude of variables and where different individual and socio-familial factors have a relevant role in academic performance, the number of adolescents with low performance and who have repeated a year is above the OECD mean. This study showed that low academic performance is related to higher levels of burnout symptoms in adolescents, in which stress management and mood factors of emotional intelligence are mediators.

Thus, failing a subject or repeating a year, which are two adverse situations in the school context, generate negative emotional states, which if not managed successfully, or if allowed to lead to prolonged worsening of the minor’s mood, can promote development of school burnout. We therefore propose the development of emotional intelligence programs for improving the response to situations that generate negative feelings, such as low performance. This could prevent and decrease school burnout in adolescents while increasing other positive variables for their academic development, such as self-esteem, interpersonal relations, classroom participation or academic wellbeing.

Similarly, emotional content should be included in educational programs directed at working on general academic competencies, as well as specific subjects using educational applications for the purpose. This could lead to significant student learning, with the consequent acquisition of personal resources for effectively coping with new academic challenges.

Finally, based on all of the above, future lines of research could study the variables involved in greater depth to find new explanatory models that contribute not only to preventing school burnout, but also to acquisition of personal resources and strategies for improving academic engagement of high school students.

## References

[1] Baños R, Baena-Extremera A, Ortiz-Camacho MM. Prediction Model of Academic Performance and Satisfaction with School According to Some Subjects of Compulsory Secondary Education. Psychol Rep. 2018;123(2): 435–451. doi: 10.1177/0033294118805004 30482113

[2] Organización para la Cooperación y el Desarrollo Económicos. Informe PISA 2018. Programa para la evaluación internacional de los estudiantes. Informe español. Madrid: Ministerio de Educación y Formación Profesional; 2019

[3] Chau K, Kabuth B, Causin-Brice O, Delacour Y, Richoux-Picard C., Verdin M, et al. Associations between school difficulties and health-related problems and risky behaviours in early adolescence: A cross-sectional study in middle-school adolescents in France. Psychiatry Res. 2016;244: 1–9. doi: 10.1016/j.psychres.2016.07.008 27455144

[4] Fiorilli C, De Stasio S, Di Chiacchio C, Pepe A, Salmera-Aro K. School burnout, depressive symptoms and engagement: Their combined effect on student achievement. Int J Educ Res. 2017;84: 1–12. doi: 10.1016/j.ijer.2017.04.001

[5] Parhiala P, Torppa M, Vasalampi K, Eklund K, Poikkeus AM, Aro T. Profiles of school motivation and emotional well-being among adolescents: Associations with math and reading performance. Learn Individ Differ. 2018;61: 196–204. doi: 10.1016/j.lindif.2017.12.003

[6] Martos Á, Pérez-Fuentes MC, Molero MM, Gázquez JJ, Simón MM, Barragán AB. Burnout y engagement en estudiantes de Ciencias de la Salud. Eur J Investig Health Psychol Educ. 2018;8(1): 23–36. doi: 10.30552/ejihpe.v8i1.223

[7] Widlund A, Tuominen H, Tapola A, Korhonen J. Gendered pathways from academic performance, motivational beliefs, and school burnout to adolescents’ educational and occupational aspirations. Learn Instr. 2020;66: 101299. doi: 10.1016/j.learninstruc.2019.101299

[8] Maganto C, Peros M, Sánchez R. El bienestar psicológico en la adolescencia: variables psicológicas asociadas y predictoras. Eur J Educ Psychol. 2019;12(2): 139–151. doi: 10.30552/ejep.v12i2.279

[9] Oliveira LS, Valente JY, Bertini C, Andreoni S, Sanchez ZM. Binge drinking and frequent or heavy drinking among adolescents: prevalence and associated factors. J Pediatr (Rio J). 2020;96(2): 193–201. doi: 10.1016/j.jped.2018.08.005 30316810 PMC9432035

[10] Li J, Hesketh T. Prevalence, risk factors, and psychosomatic symptoms of bullying in Chinese adolescents in three provinces: a cross-sectional study. The Lancet. 2019;394(1): S6. doi: 10.1016/S0140-6736(19)32342-6

[11] Li P, Zhou N, Zhang Y, Xiong Q, Nie R, Fang X. Incremental Theory of Intelligence Moderated the Relationship between Prior Achievement and School Engagement in Chinese High School Students. Front Psychol. 2017;8:1703. doi: 10.3389/fpsyg.2017.01703 29021772 PMC5623707

[12] Tortosa BM, Pérez-Fuentes MC, Molero MM, Soriano JG, Oropesa NF, Simón MM, et al. Engagement académico e Inteligencia Emocional en adolescentes. Eur J Develop Educa Psychop. 2020;8(1): 111–122. doi: 10.30552/ejpad.v8i1.136

[13] Salamon R. A 10-Year Prospective Study of Socio-Professional and Psychological Outcomes in Students from High-Risk Schools Experiencing Academic Difficulty. Front Psychol. 2020;11:1742. doi: 10.3389/fpsyg.2020.01742 32760334 PMC7372088

[14] Salmela-Aro K. Dark and bright sides of thriving–School burnout and engagement in the Finnish context. Eur J Dev Psychol. 2017;14: 337–349. doi: 10.1080/17405629.2016.1207517

[15] Schaufeli WB, Martínez IM, Pinto AM, Salanova M, Bakker AB. Burnout and engagement in university students: A crossnational study. J Cross Cult Psychol. 2002;33(5): 464–481. doi: 10.1177/0022022102033005003

[16] Cadime I, Marques A, Lima S, Rego S, Pereira J, Ribeiro I. Well-being and academic achievement in secondary school pupils: The unique effects of burnout and engagement. J Adolesc. 2016;53: 169–179. doi: 10.1016/j.adolescence.2016.10.003 27814494

[17] Palos R, Maricutoiu LP, Coste I. Relations between academic performance, student engagement and student burnout: A cross-lagged analysis of a two-wave study. Stud Educ Evaluation. 2019;60: 199–204. doi: 10.1016/j.stueduc.2019.01.005

[18] Chen T, Liu LL, Cui JF, Chen XJ, Wang Y. Developmental trajectory of time perspective: From children to older adults. PsyCh J. 2016;5(4): 245–255. doi: 10.1002/pchj.140 27718341

[19] Kinnunen JM, Lindfors P, Rimpelä A, Salmela-Aro K, Rathmann K, Perelman J, et al. Academic well-being and smoking among 14- to 17-year-old schoolchildren in six European cities. J Adolesc. 2016;50: 56–64. doi: 10.1016/j.adolescence.2016.04.007 27208481

[20] May RW, Bauer KN, Seibert GS, Jaurequi ME, Ficham FD. School burnout is related to sleep quality and perseverative cognition regulation at bedtime in young adults. Learn Individ Differ. 2020;78: 101821. doi: 10.1016/j.lindif.2020.101821

[21] Engels MC, Pakarinen E, Lekkanen MK, Verschueren K. Students’ academic and emotional adjustment during the transition from primary to secondary school: A cross-lagged study. J Sch Psychol. 2019;76: 140–158. doi: 10.1016/j.jsp.2019.07.012 31759462

[22] Scott-Parker B. Emotions, behaviour, and the adolescent driver: A literature review. Traffic Psychol Behav. 2017;50: 1–37. doi: 10.1016/j.trf.2017.06.019

[23] Brackett MA, Rivers SE, Salovey P. Emotional Intelligence: Implications for Personal, Social, Academic, and Workplace Success. Soc Personal Psychol Compass. 2011;5(1): 88–103. doi: 10.1111/j.1751-9004.2010.00334.x

[24] Salovey P, Mayer JD. (1990). Emotional intelligence. Imagin Cogn Pers. 1990;9: 185–211. doi: 10.1080/00223891.1990.9674037 2348356

[25] Pérez-Fuentes MC, Molero MM, Barragán AB, Gázquez JJ. Family Functioning, Emotional Intelligence, and Values: Analysis of the Relationship with Aggressive Behavior in Adolescents. Int J Environ Res Public Health. 2019;16(3): 478. doi: 10.3390/ijerph16030478 30736326 PMC6388189

[26] Durlak JA, Weissberg RP, Dymnicki AB, Taylor RD, Schellinger K.B. The Impact of Enhancing Students’ Social and Emotional Learning: A Meta‐Analysis of School‐Based Universal Interventions. Child Dev. 2011;82(1): 405–432. doi: 10.1111/j.1467-8624.2010.01564.x 21291449

[27] de la Barrera U, Mónaco E, Postigo-Zegarra S, Gil-Gómez J-A, Montoya-Castilla I. EmoTIC: Impact of a game-based social-emotional programme on adolescents. PLoS ONE. 2021;16: e0250384. doi: 10.1371/journal.pone.0250384 33861813 PMC8051799

[28] Moeller J, Brackett MA, Ivcevic Z, Whitte AE. High school students’ feelings: Discoveries from a large national survey and an experience sampling study. Learn Instr. 2020;66: 101301. doi: 10.1016/j.learninstruc.2019.101301

[29] Wang MT, Chow A, Hofkens T, Salmela-Aro K. The trajectories of student emotional engagement and school burnout with academic and psychological development: Findings from Finnish adolescents. Learn Instr. 2015;36: 57–65. doi: 10.1016/j.learninstruc.2014.11.004

[30] Leigh E, Chiu K, Clark DM. Is concentration an indirect link between social anxiety and educational achievement in adolescents? PLoS ONE, 2021;16: e0249952. doi: 10.1371/journal.pone.0249952 33989297 PMC8121284

[31] Ninivaggi FJ. Chapter 3—Emotional Intelligence and Mindfulness. En Ninivaggi FJ, editor. Learned Mindfulness. Psysician Engagement and M.D. Wellness. Academic Press; 2020. pp. 47–71.

[32] Pérez-Fuentes MC, Molero MM, Gázquez JJ, Simón MM. Analysis of Burnout Predictors in Nursing: Risk and Protective Psychological Factors. Eur J Psychol Appl to Leg Context. 2019;11(1): 33–40. doi: 10.5093/ejpalc2018a13

[33] Schoeps K, Tamarit A, de la Barrera U, González R. Effects of emotional skills training to prevent burnout syndrome in schoolteachers. Ansiedad y Estrés. 2019;25(1): 7–13. doi: 10.1016/j.anyes.2019.01.002

[34] Enríquez H, Ramos N, Esparza O. Impact of the Mindful Emotional Intelligence Program on emotional regulation in college students. Int Psicol Ter Psicol. 2017;17(1): 39–48. doi: 10.1037/t05430-000

[35] Prado V, Villanueva L, Górriz A. Trait emotional intelligence and subjetive well-being in adolescents: the moderating role of feelings. Psicothema. 2018;30(3): 310–315. doi: 10.7334/psicothema2017.232 30009754

[36] Van Loon AWG, Creemers HE, Beumer WY, Okorn A, Vogelaar S, Saab N, et al. Can Schools Reduce Adolescent Psychological Stress? A Multilevel Meta-Analysis of the Effectiveness of School-Based Intervention Programs. J Youth Adolesc. 2020;49: 1127–1145. doi: 10.1007/s10964-020-01201-5 32034632 PMC7237523

[37] Lei H, Cui Y. Effects of Academic Emotions on Achievement among mainland chinese students: a meta-analysis. Soc Behav Pers. 2016;44(9): 1541–1553. doi: 10.2224/sbp.2016.44.9.1541

[38] Piryaei S, Mohebbi M, Khademi M, Khademi E. Academic stress and emotion regulation in the Iranian female students with high and low academic performance. Eur Psychiat. 2017;41: S787–S788. doi: 10.1016/j.eurpsy.2017.01.1506

[39] Puertas-Molero P, Zurita-Ortega F, Chacón-Cuberos R, Castro-Sánchez M, Ramírez-Granizo I, González-Valero G. Emotional intelligence in the field of education: a meta-analysis. Ann Psychol. 2020;36(1): 84–91. doi: 10.6018/analesps.345901

[40] Baytemir L. (2019). Experiences of School as a Mediator between Interpersonal Competence and Happiness in Adolescents. Ann Psychol. 2019;35(2): 259–268. doi: 10.6018/analesps.35.2.320311

[41] Gómez-Ortiz O, Romera EM, Jiménez-Castillejo R, Ortega-Ruiz R, García-López LJ. Parenting practices and adolescent social anxiety: A direct or indirect relationship? Int J Clin Health Psychol. 2019;19(2): 124–133. doi: 10.1016/j.ijchp.2019.04.001 31193117 PMC6517642

[42] Pérez-Fuentes MC, Molero MM, Simón MM, Oropesa NF, Gázquez JJ. Validation of the Maslach Burnout Inventory-Student Survey in spanish adolescents. Psicothema. 2020;32(3): 444–451. doi: 10.7334/psicothema2019.373 32711681

[43] Pérez-Fuentes MC, Gázquez JJ, Mercader I, Molero MM. Brief Emotional Intelligence Inventory for Senior Citizens (EQ-i-M20). Psicothema. 2014;26(4): 524–530. doi: 10.7334/psicothema2014.166 25340901

[44] JASP Team. JASP (Version 0.11.1) [Computer software]. 2019

[45] Rosseel Y. Lavaan: An R package for structural equation modeling and more. Version 0.5–12 (BETA). J Stat Softw, 2012;48(2): 1–36.

[46] Biesanz JC, Falk CF, Savalei V. Assessing Mediational Models: Testing and Interval Estimation for Indirect Effects. Multivariate Behav Res. 2010; 45(4): 661–701. doi: 10.1080/00273171.2010.498292 26735714

[47] McDonald R.P. Test theory: A unified approach. Mahwah, NJ: Lawrence Erlbaum Associates; 1999

[48] Ventura-León JL, Caycho T. El coeficiente Omega: un método alternativo para la estimación de la confiabilidad. Rev. Latinoam. Cienc. Soc. Niñez Juv. 2017;15: 625–627.

[49] Szczygiel DD, Mikolajczak M. Emotional Intelligence Buffers the Effects of Negative Emotions on Job Burnout in Nursing. Front. Psychol. 2018; 9. doi: 10.3389/fpsyg.2018.02649 30627113 PMC6309155

